# Role of Intramuscular Fat and Lean Muscle in Surface Electromyography Amplitude of the Gluteus Medius in Older Adults

**DOI:** 10.1093/geroni/igaa057.417

**Published:** 2020-12-16

**Authors:** Marcel Lanza, Vicki Gray, Alice Ryan, Will Perez, Odessa Addison

**Affiliations:** 1 University of Maryland, Baltimore, Baltimore, Maryland, United States; 2 University of Maryland School of Medicine, Baltimore, Maryland, United States; 3 Baltimore VA Medical Center, Baltimore, Maryland, United States

## Abstract

Surface electromyography (sEMG) is frequently used to assess muscle activation in older individuals. Subcutaneous fat is one well-known factor that influences sEMG amplitude. The amount of intramuscular fat (IMAT) may negatively impact the muscles ability to produce force with aging, while high density lean tissue (HDL; fat free muscle) has an opposite effect. However, influence of IMAT or HDL on sEMG amplitude remains unclear. Thus, the aim was to investigate the influence of IMAT and HDL on sEMG amplitude of the gluteus medius (GM) muscle during a maximal voluntary isometric contraction (MVIC) in older adults. Twelve older adults (7 females; age: 71±3 y; BMI= 29±4 Kg/m2; X ± SD) underwent a CT scan to determine IMAT and HDL cross-sectional area in the GM. IMAT and HDL were normalized as a percentage of the total muscle area. Maximal hip abduction MVIC was measured at 30□ hip abduction in standing, while sEMG was recorded from the GM muscle. Spearman correlations showed a positive association between GM HDL and sEMG amplitude (r = 0.692, P = 0.013) and negative between GM IMAT and sEMG amplitude (r = -0.683, P = 0.014). This is the first study to demonstrate the amount of IMAT may limit the ability to activate the hip abductor muscle. Given that muscle activation is a determinant of strength, interventions to lower levels of IMAT and increase levels of lean muscle may be important to slowing decreases in strength with aging.

